# Removal of Pharmaceutical Residues by Ferrate(VI)

**DOI:** 10.1371/journal.pone.0055729

**Published:** 2013-02-07

**Authors:** JiaQian Jiang, Zhengwei Zhou

**Affiliations:** School of Engineering and Built Environment, Glasgow Caledonian University, Glasgow, Scotland, United Kingdom; National University of Singapore, Singapore

## Abstract

**Background:**

Pharmaceuticals and their metabolites are inevitably emitted into the waters. The adverse environmental and human health effects of pharmaceutical residues in water could take place under a very low concentration range; from several µg/L to ng/L. These are challenges to the global water industries as there is no unit process specifically designed to remove these pollutants. An efficient technology is thus sought to treat these pollutants in water and waste water.

**Methodology/Major Results:**

A novel chemical, ferrate, was assessed using a standard jar test procedure for the removal of pharmaceuticals. The analytical protocols of pharmaceuticals were standard solid phase extraction together with various instrumentation methods including LC-MS, HPLC-UV and UV/Vis spectroscopy. Ferrate can remove more than 80% of ciprofloxacin (CIP) at ferrate dose of 1 mg Fe/L and 30% of ibuprofen (IBU) at ferrate dose of 2 mg Fe/L. Removal of pharmaceuticals by ferrate was pH dependant and this was in coordinate to the chemical/physical properties of pharmaceuticals. Ferrate has shown higher capability in the degradation of CIP than IBU; this is because CIP has electron-rich organic moieties (EOM) which can be readily degraded by ferrate oxidation and IBU has electron-withdrawing groups which has slow reaction rate with ferrate. Promising performance of ferrate in the treatment of real waste water effluent at both pH 6 and 8 and dose range of 1–5 mg Fe/L was observed. Removal efficiency of ciprofloxacin was the highest among the target compounds (63%), followed by naproxen (43%). On the other hand, n-acetyl sulphamethoxazole was the hardest to be removed by ferrate (8% only).

**Conclusions:**

Ferrate is a promising chemical to be used to treat pharmaceuticals in waste water. Adjusting operating conditions in terms of the properties of target pharmaceuticals can maximise the pharmaceutical removal efficiency.

## Introduction

Pharmaceuticals such as antibiotics, anti-inflammatory drugs, β-blockers and X-ray contrast media are widely used in our daily life. These pharmaceuticals and their metabolites cannot be fully utilized by human beings or animals and are inevitably emitted into the waters by execration [1]–[4] and/or through the discharge of industry effluents and hospitals waste waters [5], [6]. Results of toxicology studies have revealed that some pharmaceuticals are suspected to have direct toxicity to certain aquatic organisms [7]–[9] and they could accumulate slowly, and finally lead to irreversible change on wildlife and human beings [10]. The adverse environmental and human health effects could take place under a very low concentration range; from several µg/l to ng/L. Due to this, pharmaceutical and personal care products (PPCPs) and endocrine disrupting chemicals (EDCs) are classified as emerging micro-pollutants which have been a significant issue of environmental and public health concern.

The presence of emerging micro pollutants and their potential toxicity are challenge to the global water industries as there is no unit process specifically designed to remove these pollutants; activated sludge and secondary sedimentation in most wastewater treatment works (WWTWs) seems to be inefficient to eliminate them [11]–[15]. Thus, a number of recent studies have been carried out to explore suitable technologies to treat pharmaceutical residuals from water and wastewater. Ozonation was found to be effective to remove pharmaceuticals in municipal WWTWs [16]. Nanofiltration (NF) and reverse osmosis (RO) membrane filtration have been applied at bench, pilot and full scale [17]. Activated carbon adsorption [18] has also been proved as an efficient process to remove pharmaceuticals; addition of 5 mg/L of powder activated carbon with a 4-h contact time removed 50% to >98% of the volatile PPCPs and 10% to >95% of the polar PPCPs [19].

Another promising technique which can address the concerns on pharmaceutical residues is ferrate (VI) (Fe^VI^O_4_^2−^, Fe (VI)). Ferrate (VI) exhibits many advantages because of its dual function as an oxidant and a coagulant [20]–[24] and it has green chemical properties [25]. Ferrate therefore has been successfully applied into water remediation processes [26]–[30] and oxidation of carbohydrates [31] and nitrogen-containing pollutants [32]. And researches on the removal of pharmaceuticals and other micro pollutants by ferrate (VI) have been conducted recently (e.g., [33]–[38]). Moreover, ferrate (VI) is capable of removing more than 85% of various micro-pollutants containing electron-rich moieties (ERM) [39].

This paper aims to address the issue of the performance optimisation of pharmaceutical treatment by ferrate (VI). The dose of ferrate, test water pHs and the physical and chemical properties of pharmaceuticals are major factors to influence the overall treatment efficiency and these were studied in this work.

## Materials and Methods

### Chemicals and Reagents

Ciprofloxacin (98%, HPLC grade), ibuprofen (98%, GC grade), ibuprofen sodium salt (IBU-Na) and potassium ferrate (VI) (>90%) were purchased from Sigma-Aldrich; ciprofloxacin hydrochloride BioChemica (CIP·HCl) was purchased from VWR; other chemicals and reagents used were obtained from Fisher Scientific. All chemicals and reagents were used without further purification. Deionised water used was generated by Elgacan B114 deioniser.

### Ferrate Working Solution and Model Test Solution

The ferrate (VI) working solution (1 g/L) was prepared by the addition of solid K_2_FeO_4_ to 0.005 M Na_2_HPO_4_/0.001 M borate buffer solution at pH 9.0, the pH at which the ferrate solutions are most stable [40].

Both CIP and IBU stock solutions were prepared separately in deionised water with the concentration of 10 mg/L. Several types of model test solution samples (Table 1) were prepared by the dilution of stock solutions to 1 L with tap water, with the solution pH (6.8–7.3) unadjusted before dosing ferrate (VI).

**Table 1:** Model test solutions and their specific analytical methods.

No	Model water samples	Analytical methods
1	100 µg/L IBU	Oasis HLB SPE+LC-MS
2	10 µg/L IBU	Oasis HLB SPE+LC-MS
3	100 µg/L CIP	Strata-X SPE+UV/Vis
4	10 µg/L CIP	Strata-X SPE+HPLC-UV
5	10 µg/L IBU +10 µg/L CIP	Strata-X SPE+HPLC-UV

### Real Wastewater Effluents and Target Pharmaceuticals

The wastewater samples were taken from the second sedimentation effluent at Shieldhall WWTW, Glasgow, and the major properties of the samples were COD = 35 mg/L, turbidity = 2 NTU and pH was 7.37.

Two litres of raw samples were analysed for pharmaceutical concentrations, and the sample was then spiked with selected pharmaceuticals; each with concentration of 10 µg/L. The spiked pharmaceuticals are as follows, 1) *X-ray contrast media*: Iopamidol, Ammonium diatrizoate; 2) *Antibiotics*: Ciprofloxacin, Sulphamethoxazole, N-acetyl sulphamethoxazole, Erythromycin-H_2_O; 3) *Non-steroidal anti-inflammatory druds* (*NSAID)*: Naproxen, Ibuprofen; 4) *β-blockers:* Atenolol; 5) *Antineoplasic*: Cyclophosphamide, Ifosfamide; 6) *Antiepileptics*: Carbamazepine; 7) *Blood Lipid Lowering*: Bezafibrate and 8) *Local anesthetic*: Lidocaine.

### Jar Test

A series of jar test experiments was carried out with a six-unit stirrer (Kemiraflocculator 2000, Kemwater) under the following protocol: fast mixing for 1 min at 400 rpm; slow mixing for 20 min at 40 rpm; and then sedimentation for 60 min. The ferrate dose applied was 0–4 mg/L as Fe, and pH of solutions was adjusted by 0.1 M H_2_SO_4_ or 0.05 M NaOH to 7.0–7.5. All experiments were duplicated. For the real wastewater effluents, the jar test protocol was the same as the above stated but ferrate dose sued was 0, 1, 2, 3, 4, and 5 mg/L as Fe and working pH was pH 6 and 8.

### Analytical Methods

As shown in Table 1, three analytical methods were employed for different model test samples. Specifically, for model test solution containing IBU only, solid phase extraction (SPE, Oasis HLB cartridges)+liquid chromatography (LC)-mass spectrometry (MS) was employed, which was conducted in the analytical lab of Bodensee-Wasserversorgung (BWV), Germany. In addition, a simple analytical method, SPE+UV/Vis spectrophotometry, was applied to 100 µg/L CIP model water samples. Moreover, for 10 µg/L CIP and mixed 10 µg/L IBU&CIP water samples, SPE+high performance liquid chromatography (HPLC)-UV was proposed.

#### Solid phase extraction

The treated model test solution samples were filtered through 0.45 µm cellulose nitrate membrane filters (Milipore) and then enriched by solid phase extraction (SPE). Two types of SPE cartridges were employed in the experiment: Strata-X (200 mg/6 mL and 1 g/20 mL, from Phenomenex) and Oasis HLB (3 mL/60 mg, from Waters USA). Oasis HLB cartridges were only used for those model water samples containing IBU only. Generally, for Strata-X 200 mg/6 mL cartridges, the extraction method was: (1) condition: 4 mL methanol (MeOH); (2) equilibrate: 4 mL deionised water; (3) loading samples: desired amount of model water samples under vacuum at a flow rate of 5–10 mL/min; (4) wash: 2 mL 50∶50 (v/v) MeOH/H_2_O; (5) dry: 15 min under gentle nitrogen flow; and (6) elute: 2×2 mL 2∶49:49 (v/v/v) formic acid/MeOH/ACN. Elutes were either re-constituted to 5 mL for UV/Vis spectrophotometric measurement or dried down to 1 mL for HPLC-UV detection.

On the other hand, IBU only model test solutions were extracted by Oasis HLB cartridges with the use of SPE preparation system Gilson GX-271 ASPEC. The programmed SPE procedure was as follows: (1) condition: 3 mL methanol and 3 mL H_2_O; (2) loading samples: desired amount of water samples at 10 mL/min; (3) wash: 0.5 mL water; (4) dry: 10 min under a gentle stream of nitrogen gas; and (5) elute: 2×2 mL methanol. Then, samples elutes were evaporated to less than 1 mL using nitrogen gas by Barkey sample evaporation system (Barkey, Germany), and reconstituted with methanol to 1 mL for LC/MS analysis. Prior to extraction, selected samples were spiked with 25 µL of thidiazuron solution (20 ng/µL) as an internal standard.

For the real wastewater treatment, the samples were filtered by 1.2 µm glass fibre filter papers (Fisher), the filtrates were then filtered by 0.45 µm membrane filters (Milipore). The Tandem SPE cartridges used were Strata-X 1 g/12 mL+ENV+500 mg/6 mL, and the extraction procedures were: 1)Adjusted the pH of solution to 2 by 2 M H_2_SO_4_, spiked with 1 mL deuterated internal standards; 2) 10 mL methanol +10 mL water were added for the conditioning; 3) flow rate was 10 mL/min for loading samples; 4) 10 mL water was used for washing samples and 5) Drying the cartridges under N_2_ flow. After extraction, the SPE cartridges were labelled and kept in the freezer for future elution.

The elution was conducted on a Phenomenex SPE 24-position vacuum manifold. The solvents used for the elution are shown in Table 2, and all fractions are collected in silanised vials, combined and dried down at 50°C under a stream of N_2_.

**Table 2:** The solvents used for the elution.

Cartridge	Water	0.1% Formic acid in ACN/MeOH (50/50)
Strata-X	3×4 ml	4×4 ml
ENV+	3×2 ml	4×2 ml

#### UV/Vis spectrophotometry

The concentrations of CIP in the 5-mL Strata-X SPE elutes could be measured by a UV/Vis spectrophotometer (Jenway 6505 with 10 mm light-path) at the wavelength of 280 nm. A six-point calibration curve was generated based on absorption of standard CIP solutions in 2∶49:49 (v/v/v) formic acid/MeOH/ACN at 280 nm. Concentrations of CIP in model wastewater samples were calculated based on the absorbance reading and the established calibration curve [41].

#### HPLC-UV analysis

The HPLC separations were performed on the Agilent 1100 system (Agilent Technologies) consisting of a degasser, a binary pump, a thermostated column oven and a diode array detector (DAD). 50 µL of samples was manually injected to a 2.6 µm, 100 mm×2.10 mm reversed phase Kinetex XB-C_18_ column (Phenomenex). The column was kept at 25°C and eluted with 0.1% formic acid in deionised water and acetonitrile (ACN) at a flow rate of 0.2 mL/min. The elution started with 20% ACN and then with a linear gradient from 20% to 30% ACN over the next 3 min. Then the percentage of ACN was raised to 55% in 2 min, held at this percentage for 9 min and finally lowered to 20% in 1 min. Before the next injection, the system was allowed to equilibrate for 10 min. The DAD wavelengths for IBU and CIP detection were set at 220 nm and 280 nm, respectively.

#### LC-MS analysis

The LC-MS instrument employed to analyse IBU was Acquity LCT Premier XE system which consists of ACQUITY ultra performance liquid chromatography (UPLC) and orthogonal acceleration time-of-flight (oa-TOF) mass spectrometer (MS) (Waters, USA). The UPLC contained ACQUITY UPLC High Strength Silica (HSS) T3 Column (100 mm×2.1 mm, 1.7 µm particles, Waters, USA). The mobile phase was a mixture of two solvents (Solvent A: 0.1% formic acid in water; solvent B: 0.1% formic acid in ACN). For the analysis of IBU, the elution of the column started from initially 5% B, then a with a linear gradient increased to 90% B over the course of 7 min, then consistent till 8 min, and finally lowered back to 5% B at 8.1 min. The injection volume of sample was 250 µL, with the running time of 10 min and a flow rate of 0.3 mL/min. After LC separation, the MS analyser for IBU was ESI- mode, with the following parameters: capillary 1823.0 V, sample cone 30.0 V, desolvation temperature 350.0°C, source temp 150.0°C, and MCP detector 2600.0 V. Parent ions (MH^−^) were monitored at m/z 221.0497.

## Results and Discussion

### Analytical Methods

#### HPLC-UV

IBU and CIP were separated with a C_18_ column. The gradient elution of the column presented a clear separation of both chemicals in 15 min. CIP was eluted firstly from the column with a retention time 9.5 min. The signal of IBU at 220 nm showed a decreasing trend from 5 min and went flat again at 10.5 min. This phenomenon might be caused by the instrumental response to the elution gradient. However, the bulk of IBU came out at around 12.7 min, which was not affected by the previous decreasing signal.

The calibration of IBU and CIP were carried out simultaneously with standard solutions containing IBU and CIP. The curve covered the concentration range of 0.5–6 mg/l, and was conducted four times. The IBU calibration equation with the linear regression is y = 80.18x +0.05, where x means the concentration of IBU and y means the response of instrument, with a coefficient of correlation (r^2^) 0.997. In terms of CIP, an equation y = 28.11x−4.35 could fit the seven-point calibration well with the coefficient of correlation (r^2^) 0.998.

Recovery studies of standard solutions (n = 4) showed 78.0±14.7% of CIP and 139.1±2.2% of IBU recovered by the instrument, respectively.

#### LC-MS

A seven-point calibration curve for the concentration range of 0.04–1.2 mg/L was generated based on MS response of standard IBU solutions in methanol. Quantification of IBU was based on the response at m/z 221.0497 (MH^−^) vs. linear regression equation y = 57.76x, where x means the concentration of IBU and y means the response of the instrument, with the coefficient of correlation 0.998. Recovery was obtained by running standard IBU solutions (5 µg/L) through SPE and LC-MS process, with the mean recovery 117.7±30.6% (n = 4).

### Single Compound with Initial Concentration 100 µg/L

**Figure 1:**
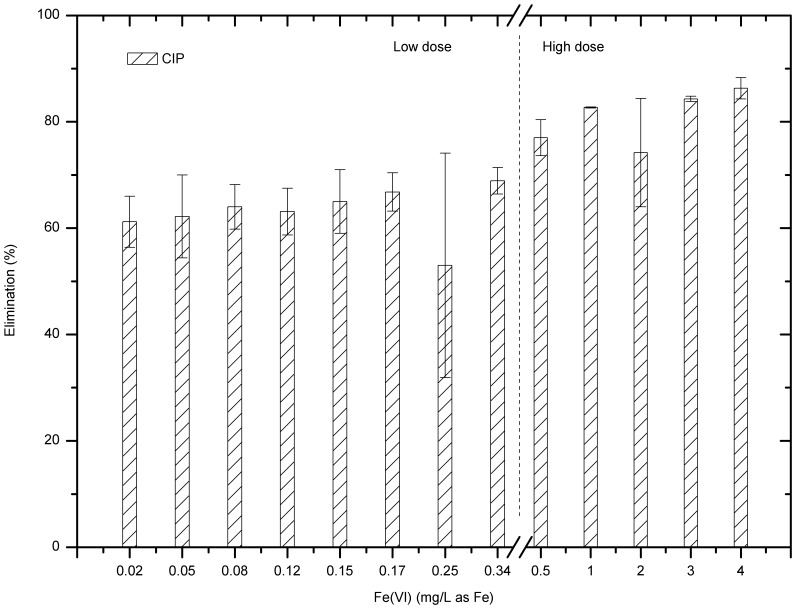
CIP removal by low doses and relatively high doses of ferrate (VI).

For CIP samples with initial concentration of 100 µg/L, average 60% of CIP was removed from the model wastewater with a low ferrate (VI) dose ranging from 0.02 to 0.34 mg/L as Fe (Fig. 1). As increasing the dose of ferrate (VI), slight improvement in the removal efficiency was observed, from 61.2% at 0.02 mg Fe/L to 68.9% at 0.34 mg Fe/L. The average removal efficiency dropped dramatically to 53.0% at dose of 0.25 mg/L as Fe may be caused by the analytical deviation. Though the overall removal of CIP at this dose range was less than 70%, the benefits of using ferrate were apparent in that low ferrate doses (maximum 0.34 mg/L as Fe) can remove up to 69% of CIP.

**Figure 2:**
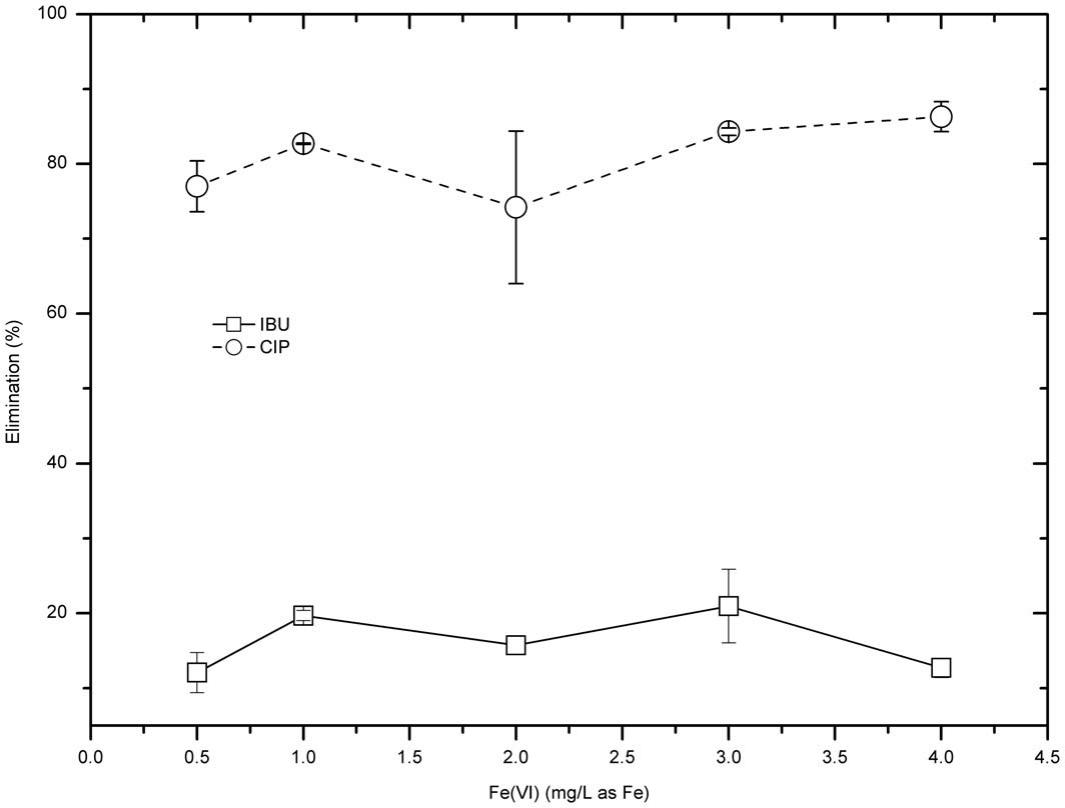
IBU and CIP removal by ferrate (VI) doses of 0.5–4 mg/L as Fe.

Further studies were carried out to investigate if relatively high ferrate doses could improve CIP removal. Figures 1 and 2 show that over 80% of CIP can be removed if the ferrate dose reached or exceeded 1 mg/L as Fe. This is consistent with that from a preliminary study [42] where a small dose of ferrate (up to 1 mg/L as Fe) can remove more than 80% of CIP and residual concentration could be lower than 1 µg/L.

However, the treating performance of IBU by ferrate (VI) was not as good as that of CIP, less than 25% of IBU was removed from the model water when dosed ferrate (VI) was 0.5–4 mg Fe/L (Fig. 2). The highest removal of IBU happened at the dosage 3 mg/L as Fe, where 21.0±4.9% of IBU was degraded.

### Single Compound with Initial Concentration 10 µg/L

**Figure 3:**
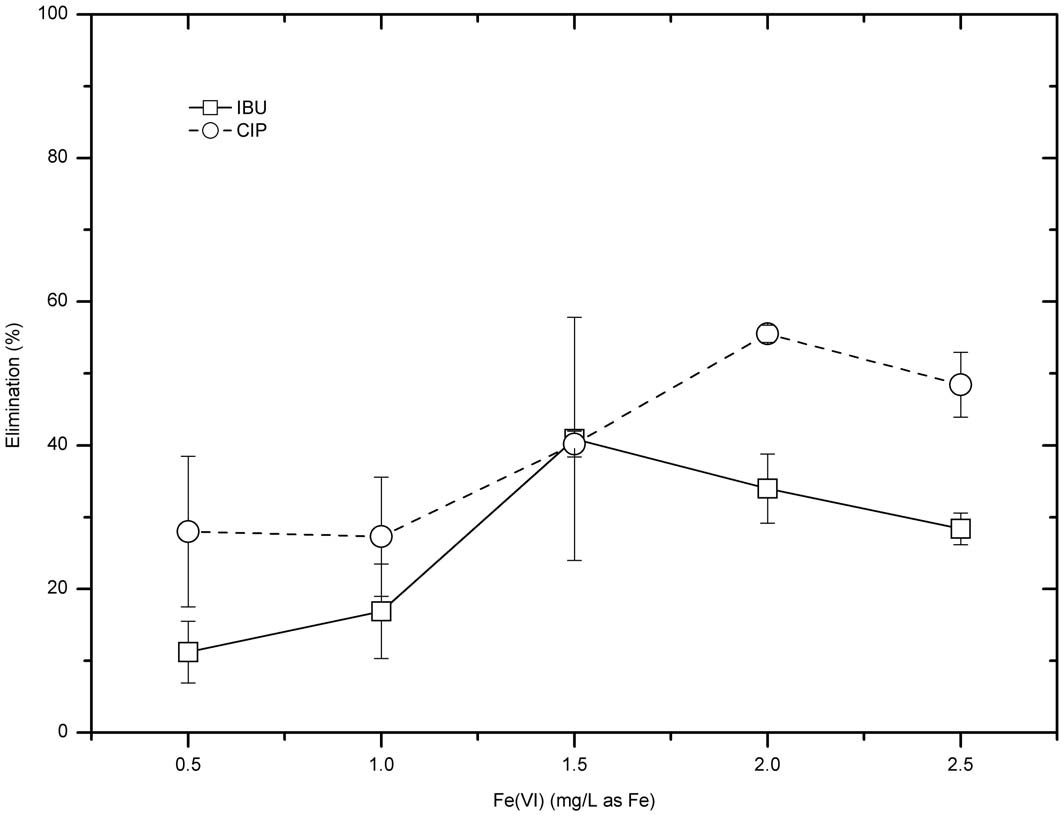
Treatment of 10 µg/L IBU model water samples by ferrate (VI) (pH = 7.05±0.25).

Compared with the case of 100 µg/L CIP model test solutions, when the initial concentration was 10 µg/L, the overall treating performance of CIP by ferrate (VI) decreased to less than 60% (Fig. 3). Specifically, when the ferrate (VI) doses were 0.5–2.5 mg Fe/L, the CIP removal efficiencies were between 25% and 60%, with a highest removal 55.5±1.2% at the dose of 2.0 mg/L as Fe.

On the other hand, the overall treating performance of IBU improved when the initial concentration was 10 µg/L. As shown in Fig. 3, the IBU removal at ferrate dose 0.5 mg Fe/L was 11.2%, which was similar to the situation when the initial IBU was 100 µg/L. Raising ferrate dose improved the IBU removal. When the dose was over 1.5 mg/L as Fe, the IBU removal could exceed 25%, with the maximum IBU removal of 40% for an optimum dose, 1.5 mg Fe/L.

### Mixed IBU&CIP

**Figure 4:**
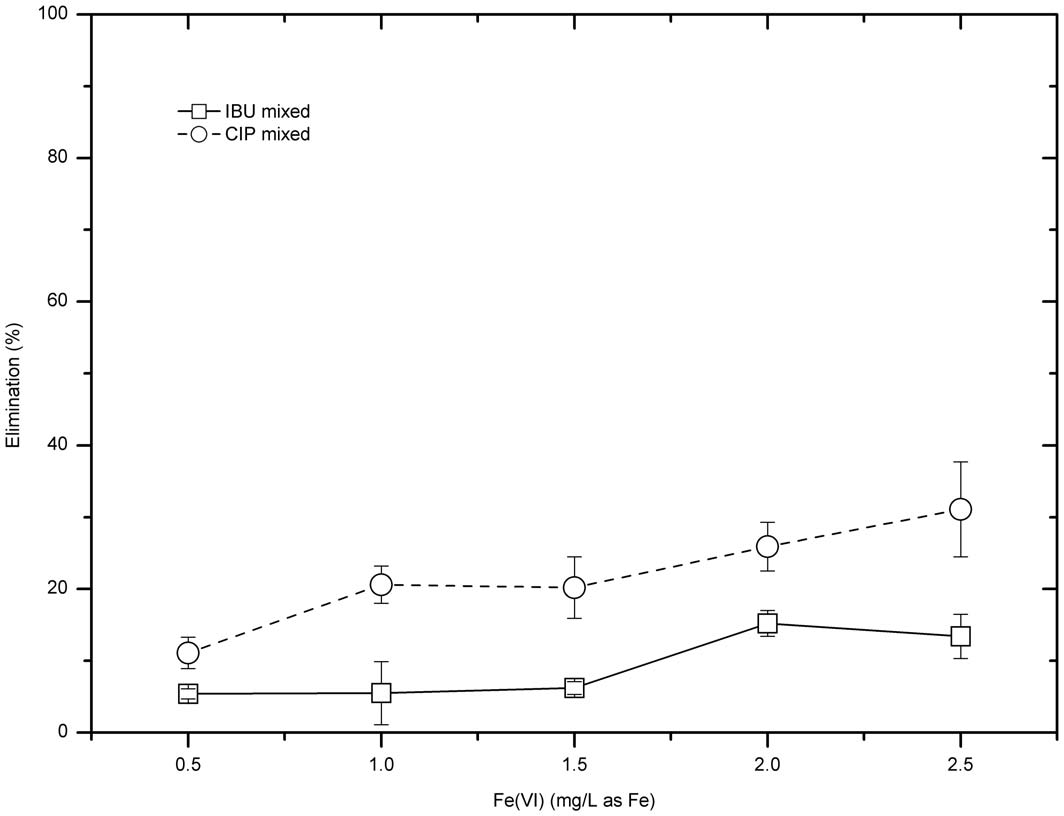
Experimental results of 10 µg/L IBU and CIP mixed model test solutions (pH = 7.05±0.25).

Further study was carried out to investigate the treating performance of mixed IBU and CIP model test solutions by ferrate. The initial concentrations of both compounds were 10 µg/L, and the ferrate doses applied were 0.5–2.5 mg/L. Results show that ferrate salts possess more capability to remove CIP than IBU (Fig. 4). Averagely, at each dose, ferrate removed CIP around 10% more than IBU. The overall removal of IBU was less than 15%, with the highest removal efficiency 15.2±1.8% at dose 2.0 mg/L as Fe. The treatment of CIP was improved by increasing the ferrate dose; when the dose increased to 2.5 mg Fe/L, the CIP removal increased to 31.1±6.6%.

**Figure 5:**
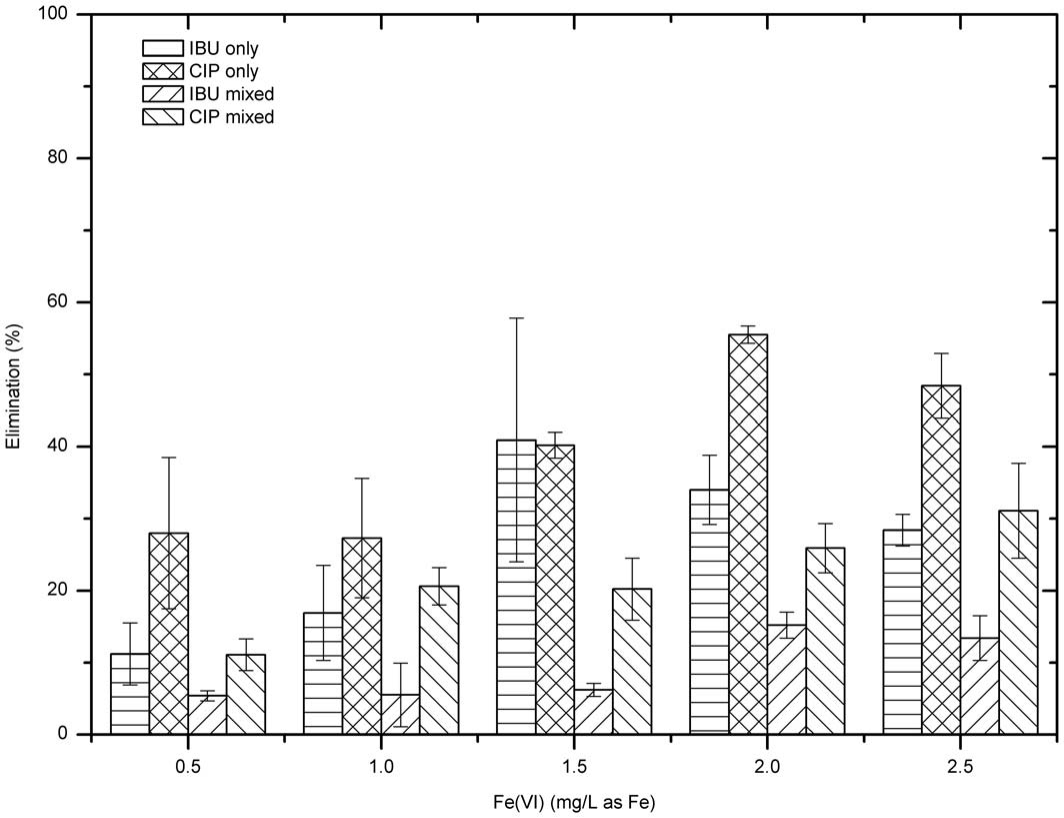
Comparison of the treating performance among different model test solutions (pH = 7.05±0.25).

Fig. 5 gives the comparison of the pharmaceutical removal among different model test solutions. Generally, the treating performance of samples with single compound was better than those with both compounds. For instance, at each dose, the CIP removal of samples with single compound was 7–30% higher than that of samples with both compounds. The biggest gap happened at dose 2.0 mg/L, where CIP removal for CIP only samples was 55.5±1.2% while for mixed samples was only 25.9±3.4%. In the case of IBU, the IBU removal in the IBU alone solutions was 5–35% higher than that of in the mixing IBU and CIP solutions.

Ferrate (VI) has shown much more capability in the degradation of CIP than that of IBU. This can be explained that CIP belongs to electron-rich organic moieties (EOM) which can be easily transformed during ferrate oxidation. However, the carboxylic group in IBU is an electron-withdrawing group with which the reactivity of ferrate is usually slow [35], [43].

### pH Dependence

**Figure 6:**
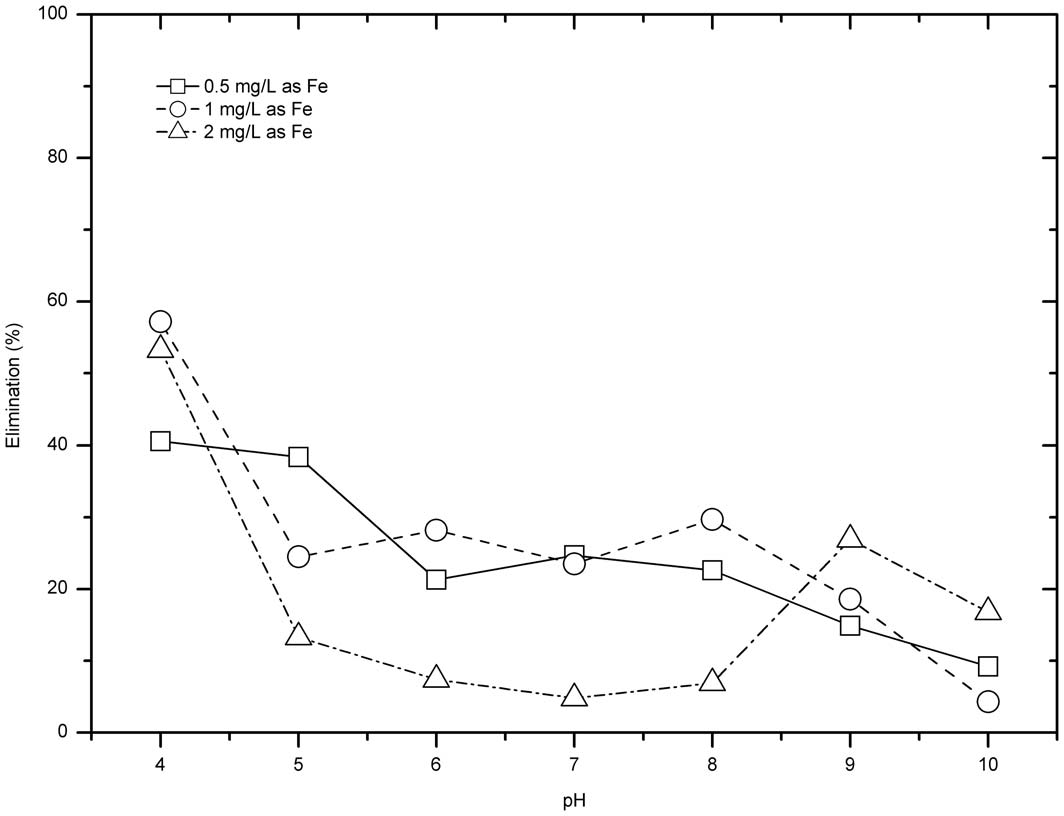
pH dependence of IBU removal by ferrate.

**Figure 7:**
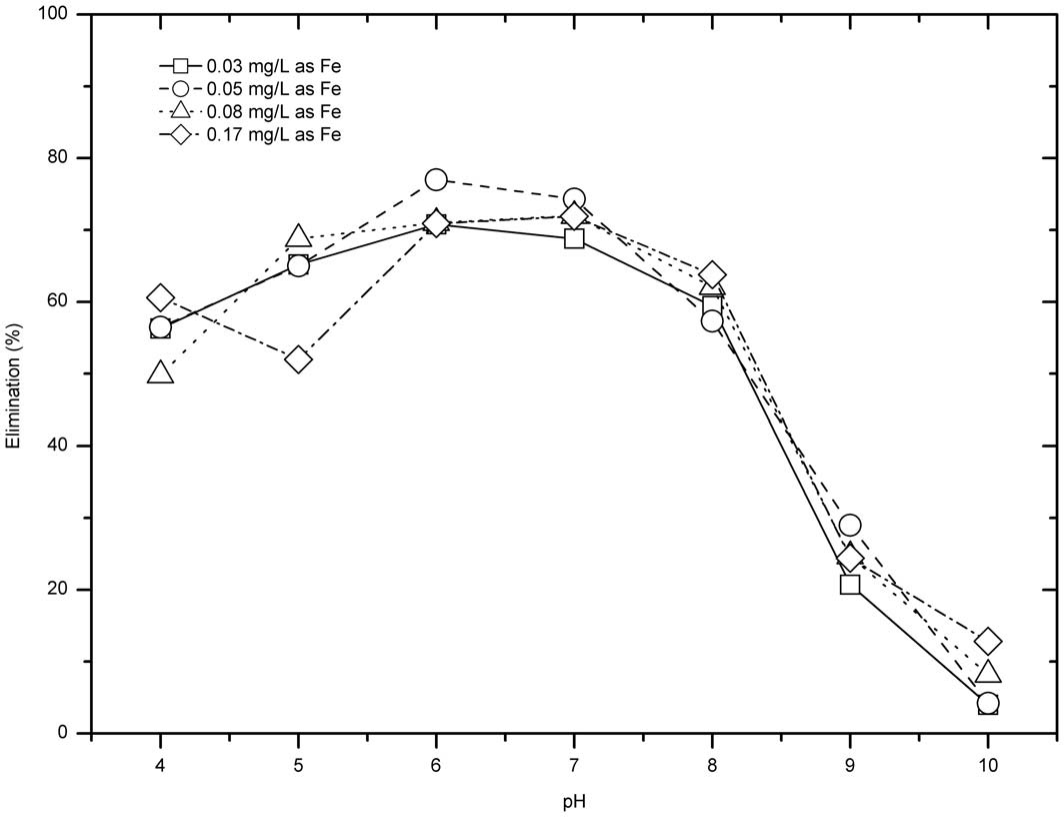
CIP removal of samples with various final pH for various ferrate doses.

Preliminary studies showed that comparing with initial pH, final solution pH after dosing ferrate has much more influence on the removal of CIP and IBU. As shown in Fig. 6, IBU removal showed great difference between pH 4 and pH 10. At pH 4, for all three doses of ferrate, IBU removal was much higher than other pH conditions, with over 50% IBU removal for the dose of 1 mg/L or 2 mg/L. However, for nearly all samples with final pH over 4, IBU removal was below 30%. As shown in Figure 7, for all ferrate doses, the CIP removal efficiencies of samples with final pH between 4 and 8 were above 45% and were much higher than those of final pH 9 and 10 (less than 25% CIP removal).

The effect of final solution pH on the IBU or CIP removal might be understood by considering compounds *pKa* values. The *pKa* of IBU is 4.91, therefore when the pH was <5, the major species would be IBU, which was relatively easy to be oxidised. For CIP, its pKa is 6.09 and 8.2, respectively, and when the pH of solution was >8, the major species of CIP would be dissociated CIP-O^−^, which was relatively difficult to be oxidised [38], and this leads to an overall decrease in CIP removal efficiency. Strong pH dependence of removal efficiency of IBU and CIP was also observed by other researchers. The reaction rate constant of ferrate (VI) versus ibuprofen (IBU) was 0.3 M^−1^ s^−1^ at pH 7.5 then slipped sharply to 0.01 M^−1^ s^−1^ at pH 9.0 [43], and the rate constant of ferrate vs. ciprofloxacin (CIP) was over 500 M^−1^ s^−1^ at pH 6 but only 10 M^−1^ s^−1^ at pH 9.5 [35]. In general, both IBU and CIP belong to electro-rich organic moieties (EOM), which can be potentiaslly transformed during ferrate oxidation and this has been demonstarted in other studies (e.g., [44]).

Strong pH effect on the IBU and CIP removal by ferrate(VI) can also be considered from ferrate(VI) speciation against solution pH. There are four ferrate(VI) species in aqueous solution that depend on pH: H_3_FeO_4_^+^, H_2_FeO_4_, HFeO_4_^−^, and FeO_4_^2−^, and the corresponding dissociation constants are pK_1_ 1.6±0.2, pK_2_ 3.5, and pK_3_ 7.23, respectively [45]. FeO_4_^2−^ is the dominant species in alkaline conditions, and HFeO_4_^−^ predominates in mildly acidic conditions. Ferrate(VI) has a higher oxidation potential at low pH (2.2 V) than in the alkaline condition (0.72 V) [23] and thus the lower the solution final pH, the stronger oxidation potential of ferrate(VI) (HFeO_4_^−^ predominates) although the stability of ferrate(VI) decreases at low pH solutions.

### Removal of Selected Pharmaceuticals Spiked in the Final Effluent by Ferrate (VI)

#### Occurrence of pharmaceuticals in the raw effluent

Six compounds were detected in the raw effluent samples, with concentrations ranging from 100 to 320 ng/L. The concentrations of naproxen, carbamazepine and atenolol were over 200 ng/L, among which naproxen exhibited the highest concentration with an average concentration 317.3 ng/L. In addition, erythromycin-H_2_O, lidocaine and bezafibrate were also detected in the effluent, with the concentrations 100–120 ng/L.

#### Removal of pharmaceuticals at pH 6 and 8

**Figure 8:**
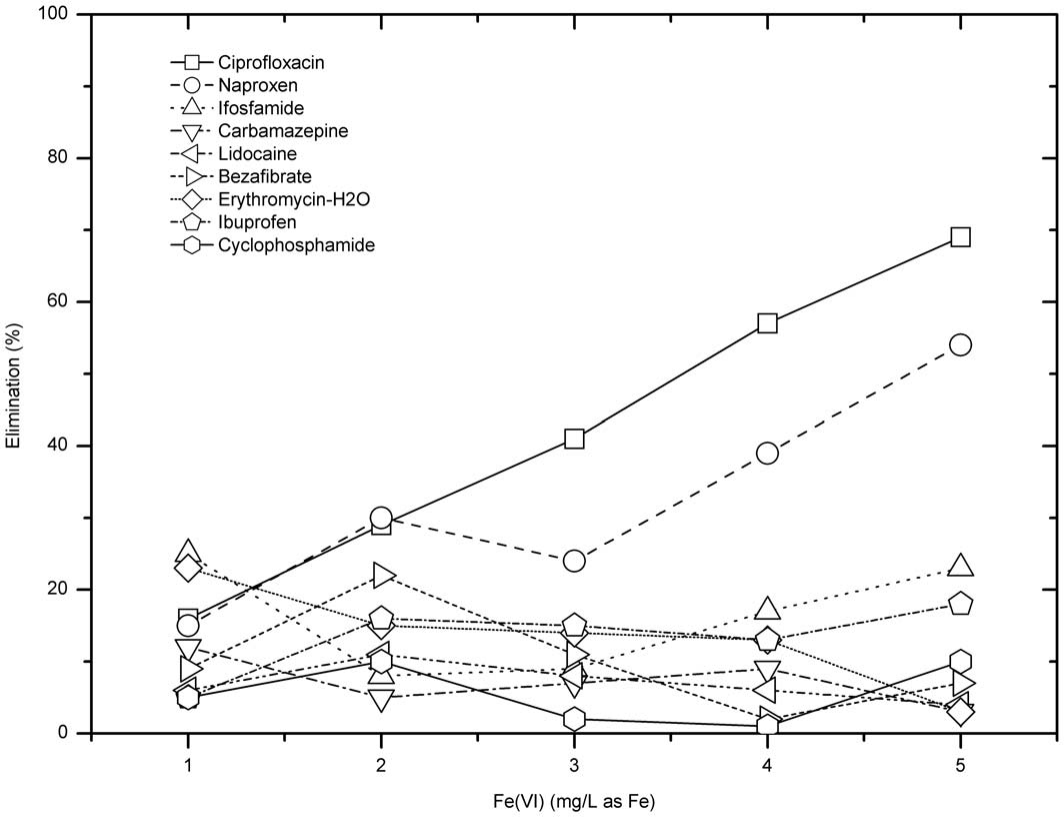
Pharmaceuticals removal by ferrate at pH 6.

As shown in Fig. 8, except ciprofloxacin and naproxen, the removal efficiencies for other compounds by ferrate were under 25% in the dosage range when the solution pH was 6. As for ciprofloxacin, the removal efficiency showed good linear correlation with the rising ferrate dose, e.g. from 16% at 1 mg/L as Fe to 69% at 5 mg/L as Fe.

**Figure 9:**
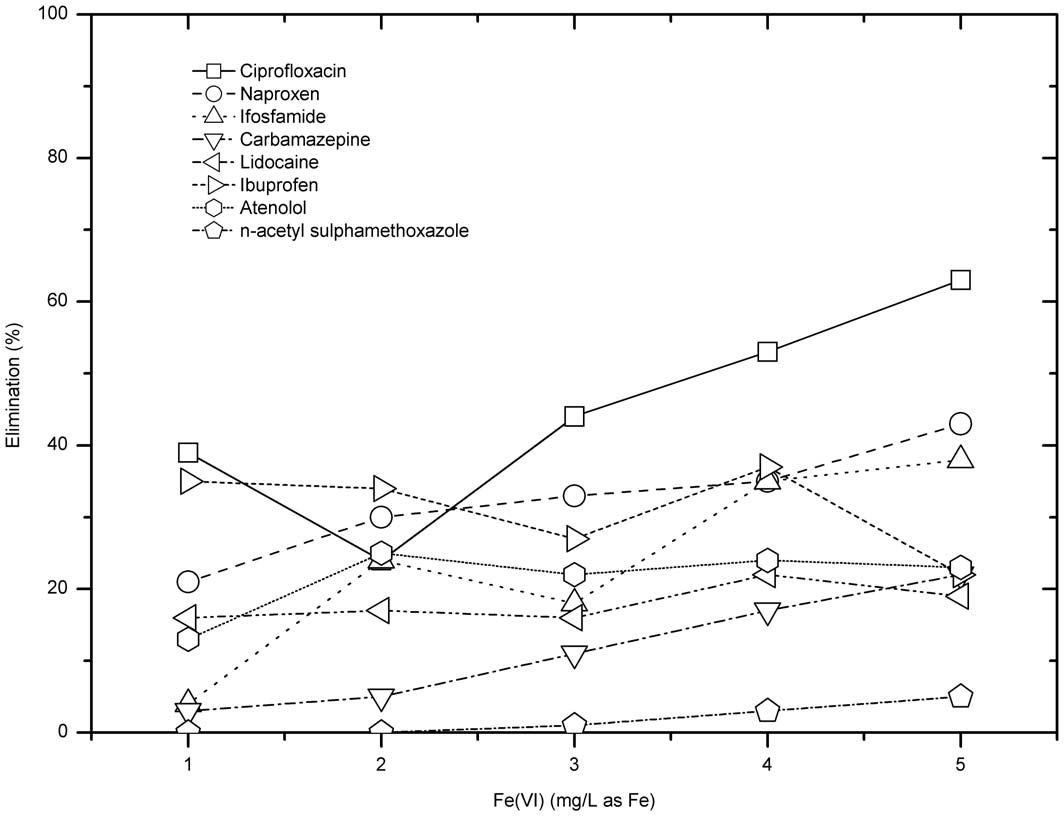
Pharmaceuticals removal by ferrate at pH 8.

Removal efficiencies of ciprofloxacin, naproxen and ibuprofen were 20–60% for ferrate doses 1–5 mg Fe/L. However, the elimination of n-acetyl sulphamethoxazole was only less than 10% for any ferrate dose. Besides, except ciprofloxacin and ifosfamide, raising ferrate dose did not improve the pharmaceutical removal significantly. As for ifosfamide and ciprofloxacin, when the ferrate dose was raised from 3 mg/L to 5 mg/L, the removal efficiency increased from 9% to 38% for ifosfamide and from 44% to 63% for ciprofloxacin, respectively (Fig. 9).

**Figure 10:**
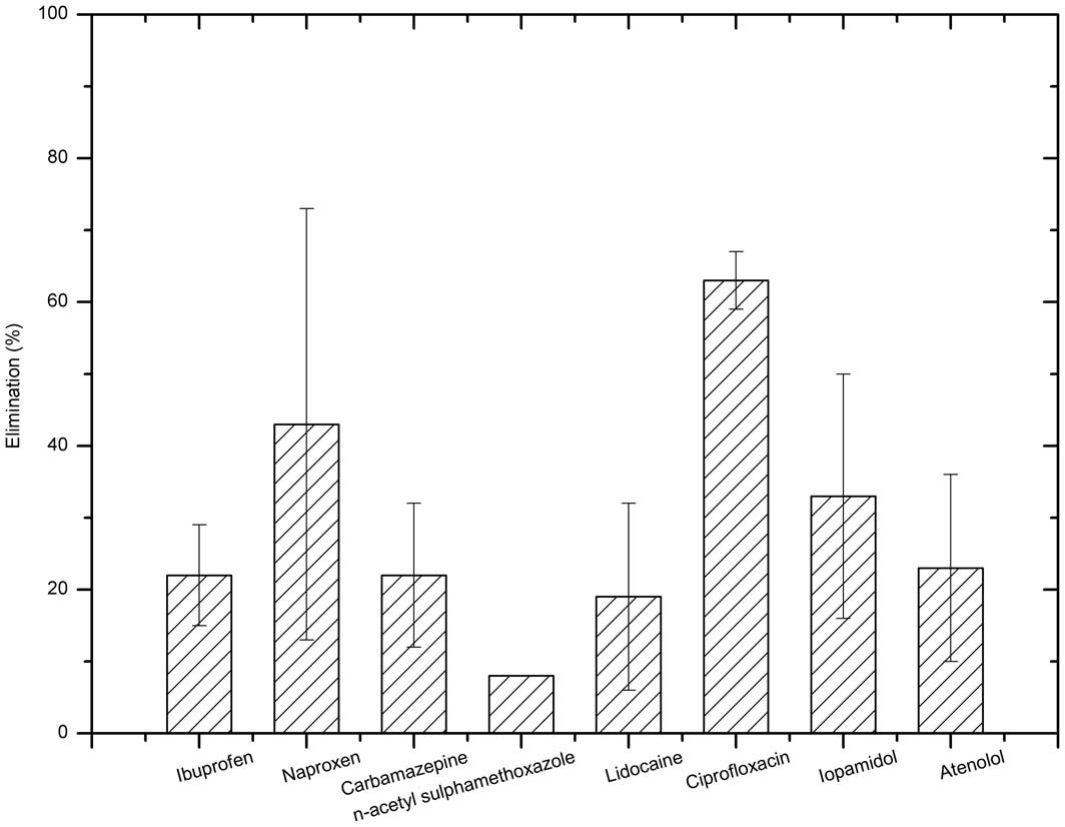
Pharmaceuticals removal by ferrate at pH 8 and ferrate dose 5 mg/L as Fe.

Fig. 10 shows the pharmaceuticals removal by ferrate when the solution pH was 8 and ferrate dose was 5 mg/L as Fe. As shown in the graph, the removal efficiency of ciprofloxacin was the highest among the compounds listed, with an average removal of 63%, followed by naproxen (43%). On the other hand, n-acetyl sulphamethoxazole was the hardest to remove by ferrate, with an average removal of 8%. The selective removal of pharmaceuticals by ferrate(VI), as demonstrated by this study, is consistent with that from other studies [35]. Ferrate(VI) can degrade electron-rich organic moieties [35], [39] of pharmaceuticals such as ciprofloxacin and then achieve high percentage removals but difficult to oxidise other pharmaceuticals such as n-acetyl sulphamethoxazole and ibuprofen. Nevertheless, sewage tertiary treatment by ferrate(VI) should achieve the removal of pharmaceuticals and other emerging micro pollutants as well as will enhance the effluent qualities in general such as lower concentrations of suspended solids, phosphate and COD [30].

### Conclusions

Results of treatment of CIP by ferrate show that ferrate can remove at least 60% of CIP from model test solutions even at very low ferrate doses (<0.3 mg/L). Besides, increasing ferrate dose up to 1 mg Fe/L as Fe can achieve more than 80% removal efficiency of CIP. However, ferrate did not show similar treating performance for IBU degradation, with only 30% IBU removal at ferrate dose of 2 mg Fe/L.

The final solution pH affects the treating performance significantly for both pharmaceuticals. When final pH of solution was greater than 8, CIP removal efficiency by ferrate drops significantly. In the case of IBU, pH 4 was the optimum pH for IBU removal by ferrate.

Ferrate has shown higher capability in the degradation of CIP than IBU. This is due to that CIP belongs to electron-rich organic moieties (EOM) which can be readily degraded by ferrate oxidation. Instead, the reactivity of ferrate with electron-withdrawing groups (carboxylic groups) in IBU is slow and consequently, lowers ferrate removal efficiency for the IBU.

Ferrate has shown promising performance at both pH 6 and 8 and dose range of 1–5 mg Fe/L in the treatment of real waste water effluent. Removal efficiency of ciprofloxacin was the highest among the target compounds, with an average removal of 63%, followed by naproxen (43%). On the other hand, n-acetyl sulphamethoxazole was the hardest to remove by ferrate, with an average removal of 8%. Furthermore, the removal efficiencies of other compounds by ferrate were between 20% and 40%.

## References

[1] Wang L, Ying GG, Zhao JL, Yang XB, Chen F, et al (2010) Occurrence and risk assessment of acidic pharmaceuticals in the Yellow River, Hai River and Liao River of north China, Science of the Total Environment. 408: 3139–3147. 10.1016/j.scitotenv.2010.04.047 20493517

[2] Comeau F, Surette C, Brun GL, Losier R (2008) The occurrence of acidic drugs and caffeine in sewage effluents and receiving waters from three coastal watersheds in Atlantic Canada, Science of the Total Environment. 396: 132–146. 10.1016/j.scitotenv.2008.02.031 18377954

[3] Nakada N, Tanishima T, Shinohara H, Kiri K, Takada H (2006) Pharmaceutical chemicals and endocrine disrupters in municipal wastewater in Tokyo and their removal during activated sludge treatment, Water Research. 40: 3297–3303. 10.1016/j.watres.2006.06.039 16938339

[4] Lishman L, Smyth SA, Sarafin K, Kleywegt S, Toito J, et al (2006) Occurrence and reductions of pharmaceuticals and personal care products and estrogens by municipal wastewater treatment plants in Ontario, Canada, Science of the Total Environment. 367: 544–558. 10.1016/j.scitotenv.2006.03.021 16697441

[5] Ternes TA (1998) Occurrence of drugs in German sewage treatment plants and rivers, Water Research. 32: 3245–3260.

[6] Lee HB, Peart TE, Svoboda ML (2007) Determination of ofloxacin, norfloxacin, and ciprofloxacin in sewage by selective solid-phase extraction, liquid chromatography with fluorescence detection, and liquid chromatography-tandem mass spectrometry, Journal of Chromatography A. 1139: 45–52. 10.1016/j.chroma.2006.11.068 17157863

[7] Ferrari B, Paxeus N, Giudice RL, Pollio A, Grric J (2003) Ecotoxicological impact of pharmaceuticals found in treated wastewaters: study of carbamazepine, clofibric acid, and diclofenac. Ecotoxicology and Environmental Safety 55 (3): 359–370. 10.1016/s0147-6513(02)00082-9 12798771

[8] Jjemba PK (2006) Excretion and ecotoxicity of pharmaceutical and personal care products in the environment. Ecotoxicology and Environmental Safety 63 (1): 113–130. 10.1016/j.ecoenv.2004.11.011 16399163

[9] Grung M, Kallqvist T, Sakshaug S, Skurtveit S, Thomas KV (2008) Environmental assessment of Norwegian priority pharmaceuticals based on the EMEA guideline. Ecotoxicology and Environmental Safety 71 (2): 328–340. 10.1016/j.ecoenv.2007.10.015 18068226

[10] Daughton CG, Ternes TA (1999) Pharmaceuticals and personal care products in the environment: agents of subtle change? Environmental Health Perspectives 107 (S6): 907–938. 10.1289/ehp.99107s6907 PMC1566206 10592150

[11] Ternes TA (1998) Occurrence of drugs in German sewage treatment plants and rivers. Water Research 32 (11): 3245–3260.

[12] Paxeus N (2004) Removal of selected non-steroidal anti-inflammatory drugs (NSAIDs), gemfibrozil, carbamazepine, beta-blockers, trimethoprim and triclosan in conventional wastewater treatment plants in five EU countries and their discharge to the aquatic environment. Water Science and Technology 50 (5): 253–260. 15497855

[13] Castiglioni S, Bagnati R, Fanelli R, Pomati F, Calamari D, et al (2006) Removal of pharmaceuticals in sewage treatment plants in Italy. Environmental Science and Technology 40 (1): 357–363. 10.1021/es050991m 16433372

[14] Nakada N, Tanishima T, Shinohara H, Kiri K, Takada H (2006) Pharmaceutical chemicals and endocrine disrupters in municipal wastewater in Tokyo and their removal during activated sludge treatment. Water Research 40 (17): 3297–3303. 10.1016/j.watres.2006.06.039 16938339

[15] Santos JL, Aparicio I, Alonso E (2007) Occurrence and risk assessment of pharmaceutically active compounds in wastewater treatment plants. A case study: Seville city (Spain). Environment International 33 (4): 596–601. 10.1016/j.envint.2006.09.014 17084895

[16] Okuda T, Kobayashi Y, Nagao R, Yamashita N, Tanaka H, et al (2008) Removal efficiency of 66 pharmaceuticals during wastewater treatment process in Japan. Water Science and Technology 57 (1): 65–71. 10.2166/wst.2008.822 18192742

[17] Comerton AM, Andrews RC, Bagley DM, Hao CY (2008) The rejection of endocrine disrupting and pharmaceutically active compounds by NF and RO membranes as a function of compound and water matrix properties. Journal of Membrane Science 313 (1–2): 323–335.

[18] Snyder SA, Adham S, Redding AM, Cannon FS, DeCarolis J, et al (2007) Role of membranes and activated carbon in the removal of endocrine disruptors and pharmaceuticals. Desalination 202: 156–181.

[19] Westerhoff P, Yoon Y, Snyder S, Wert E (2005) Fate of endocrine-disruptor, pharmaceutical, and personal care product chemicals during Simulated drinking water treatment processes. Environmental Science & Technology 39: 6649–6663. 16190224 10.1021/es0484799

[20] Jiang JQ (2007) Research progress in the use of ferrate(VI) for the environmental remediation, Journal of Hazardous Materials. 146: 617–623. 10.1016/j.jhazmat.2007.04.075 17531376

[21] Jiang JQ (2001) Development of coagulation theory and pre-polymerised coagulants for water treatment. Separation and Purification Methods 30: 127–141.

[22] Jiang JQ, Lloyd B, Grigore L (2001) Preparation and evaluation of potassium ferrate as an oxidant and coagulant for potable water treatment. Environmental Engineering Science 18: 323–328.

[23] Jiang JQ, Lloyd B (2002) Progress in the development and use of ferrate (vi) salt as an oxidant and coagulant for water and wastewater treatment. Water Research 36: 1397–1408. 11996330 10.1016/s0043-1354(01)00358-x

[24] Filip J, Yngard RA, Siskova K, Marusak Z, Ettler V (2011) Mechanisms and efficiency of the simultaneous removal of metals and cyanides by using ferrate(VI): Crucial roles of nanocrystalline iron achtungtrenung(III) oxyhydroxides and metal carbonates. Chem. Eur. J. 17: 10097–11005. 10.1002/chem.201100711 21793060

[25] Sharma VK (2002) Potassium ferrate(VI): an environmentally friendly oxidant. Adv. Environ. Res. 6(2): 143–156.

[26] Jiang JQ, Panagoulopoulos A, Bauer M, Pearce P (2006) The application of potassium ferrate for sewage treatment. Journal of Environmental Management 79(2): 215–220. 16182439 10.1016/j.jenvman.2005.06.009

[27] Jiang JQ, Wang S, Panagoulopoulos A (2007) The role of potassium ferrate(VI) in the inactivation of Escherichia coli and in the reduction of COD for water remediation. Desalination 210: 266–273.

[28] Jiang JQ, Stanford C, Alsheyab M (2009) The online generation and application of ferrate(VI) for sewage treatment-A pilot scale trial. Separation and Purification Technology 68(2): 227–231.

[29] Stanford C, Jiang JQ, Alsheyab M (2010) Electrochemical production of ferrate (iron VI): application to the wastewater treatment on a laboratory scale and comparison with iron (III) coagulant. Water, Air, and Soil Pollution 209: 483–488.

[30] Jiang JQ, Stanford C, Mollazeinal A (2012) The application of ferrate for sewage treatment: pilot- to full-scale trials, Global NEST Journal 14. (1): 93–99.

[31] Sharma VK, Sohn M, Anquandah GAK, Nesnas N (2012) Kinetics of the oxidation of sucralose and related carbohydrates by ferrate(VI). Chemosphere 87: 644–648. 22341951 10.1016/j.chemosphere.2012.01.019

[32] Sharma VK (2010) Oxidation of nitrogen-containing pollutants by novel ferrate(VI) technology: A review. J. Environ. Sci. Health, A: Toxic/Hazardous Sub. Environ. Eng. 45(6): 645–667. 10.1080/10934521003648784 20390913

[33] Jiang JQ, Yin Q, Zhou JL, Pearce P (2005) Occurrence and treatment trials of endocrine disrupting chemicals (EDCs) in wastewaters. Chemosphere 61(4): 544–550. 16202808 10.1016/j.chemosphere.2005.02.029

[34] Seitz W, Jiang JQ, Schulz W, Weber WH, Maier D, et al (2008) Formation of oxidation by-products of the iodinated X-ray contrast medium iomeprol during ozonation. Chemosphere 70(7): 1238–1246. 17892892 10.1016/j.chemosphere.2007.07.081

[35] Yang B, Ying G-G, Zhao J-L, Liu S, Zhou L-J, et al (2012) Removal of selected endocrine disrupting chemicals (EDCs) and pharmaceuticals and personal care products (PPCPs) during ferrate(VI) treatment of secondary wastewater effluents, Water Research. 46: 2194–2204. 10.1016/j.watres.2012.01.047 22342241

[36] Anquandah GAK, Sharma V, Knight AD, Batchu SR, Gardinali PR (2011) Oxidation of trimethoprim by ferrate(VI): kinetics, products, and antibacterial activity. Environ. Sci. Technol. 45: 10575–10581. 10.1021/es202237g 22032699

[37] Sharma V, Luther GW, Millero FJ (2011) Mechanisms of oxidation of organosulfur compounds by ferrate(VI). Chemosphere 82: 1083–1089. 21215423 10.1016/j.chemosphere.2010.12.053

[38] Sharma VK, Li XZ, Graham N, Doong RA (2008) Ferrate(VI) oxidation of endocrine disruptors and antimicrobials in water. Water Supply: Res. Technol. - AQUA 57(6): 419–426.

[39] Lee Y, Zimmermann SG, Kieu AT, von Gunten U (2009) Ferrate (Fe(VI)) Application for Municipal Wastewater Treatment: A Novel Process for Simultaneous Micropollutant Oxidation and Phosphate Removal, Environ. Sci. Technol. 43: 3831–3838. 10.1021/es803588k 19544895

[40] Carr JD, Kelter PB, Tabatabai A, Splichal D, Erickson J, et al.. (1985) Properties of ferrate (VI) in aqueous solution: an alternate oxidant in wastewater treatment, In Jolley, R. L. Et Al., 1285–1298.

[41] Zhou Z, Jiang JQ (2012) Detection of ibuprofen and ciprofloxacin by solid phase extraction and UV/Vis spectroscopy, J. Applied Spectroscopy 79. (3): 477–481.

[42] Jiang JQ, Zhou Z, Pahl O (2012) Preliminary study of ciprofloxacin (cip) removal by potassium ferrate(VI). Separation and Purification Technology 88: 95–98.

[43] Sharma,VK, Mishra SK (2006) Ferrate(VI) oxidation of ibuprofen: A kinetic study. Environmental Chemistry Letters 3: 182–185.

[44] Hu L, Martin HM, Arce-Bulted O, Sugihara MN, Keating KA, et al (2009) Oxidation of carbamazepine by Mn(VII) and Fe(VI): reaction kinetics and mechanism. Environ. Sci. Technol. 43: 509–515. 10.1021/es8023513 19238987

[45] Sharma VK, Burnett CR, Millero FJ (2001) Dissociation constants of the monoprotic ferrate(VI) ion in NaCl media. Phys. Chem. Chem. Phys. 3: 2059–2062.

