# Correction: Removal of Pharmaceutical Residues by Ferrate(VI)

**DOI:** 10.1371/annotation/c4b7f63f-efae-463e-88c4-ee6c47982ba0

**Published:** 2013-06-21

**Authors:** JiaQian Jiang, Zhengwei Zhou

The first author's name was misspelled. The correct name is: Jia-Qian Jiang. The correct citation is: Jiang J-Q, Zhou Z (2013) Removal of Pharmaceutical Residues by Ferrate(VI). PLoS ONE 8(2): e55729. doi:10.1371/journal.pone.0055729 

